# Applications of Manganese-Enhanced Magnetic Resonance Imaging in Ophthalmology and Visual Neuroscience

**DOI:** 10.3389/fncir.2019.00035

**Published:** 2019-05-14

**Authors:** Wenyu Deng, Muneeb A. Faiq, Crystal Liu, Vishnu Adi, Kevin C. Chan

**Affiliations:** ^1^NYU Langone Eye Center, Department of Ophthalmology, NYU School of Medicine, NYU Langone Health, New York University, New York, NY, United States; ^2^Department of Radiology, NYU School of Medicine, NYU Langone Health, New York University, New York, NY, United States; ^3^Center for Neural Science, Faculty of Arts and Science, New York University, New York, NY, United States

**Keywords:** visual pathway, manganese-enhanced magnetic resonance imaging, eye, neuroarchitecture, neuronal tract tracing, neuronal activity, glial activity

## Abstract

Understanding the mechanisms of vision in health and disease requires knowledge of the anatomy and physiology of the eye and the neural pathways relevant to visual perception. As such, development of imaging techniques for the visual system is crucial for unveiling the neural basis of visual function or impairment. Magnetic resonance imaging (MRI) offers non-invasive probing of the structure and function of the neural circuits without depth limitation, and can help identify abnormalities in brain tissues *in vivo*. Among the advanced MRI techniques, manganese-enhanced MRI (MEMRI) involves the use of active manganese contrast agents that positively enhance brain tissue signals in T1-weighted imaging with respect to the levels of connectivity and activity. Depending on the routes of administration, accumulation of manganese ions in the eye and the visual pathways can be attributed to systemic distribution or their local transport across axons in an anterograde fashion, entering the neurons through voltage-gated calcium channels. The use of the paramagnetic manganese contrast in MRI has a wide range of applications in the visual system from imaging neurodevelopment to assessing and monitoring neurodegeneration, neuroplasticity, neuroprotection, and neuroregeneration. In this review, we present four major domains of scientific inquiry where MEMRI can be put to imperative use — deciphering neuroarchitecture, tracing neuronal tracts, detecting neuronal activity, and identifying or differentiating glial activity. We deliberate upon each category studies that have successfully employed MEMRI to examine the visual system, including the delivery protocols, spatiotemporal characteristics, and biophysical interpretation. Based on this literature, we have identified some critical challenges in the field in terms of toxicity, and sensitivity and specificity of manganese enhancement. We also discuss the pitfalls and alternatives of MEMRI which will provide new avenues to explore in the future.

## Introduction

**FIGURE 1:**
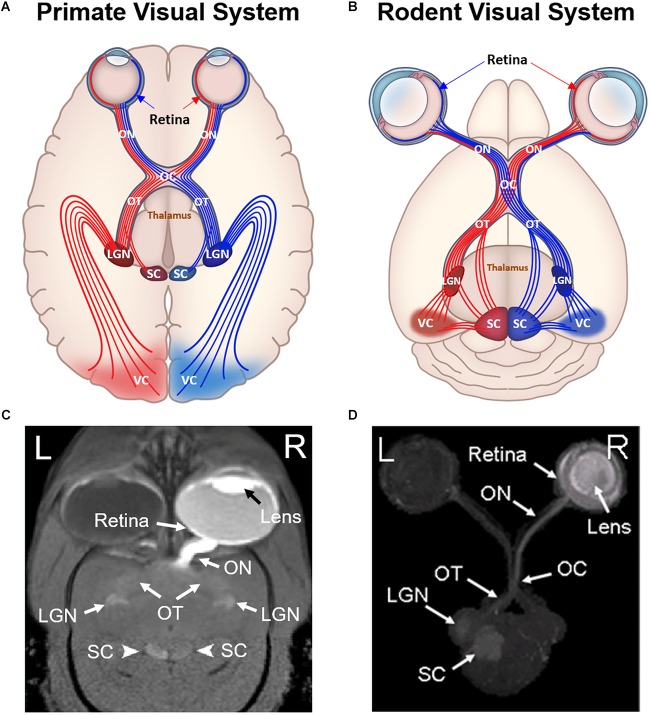
Comparisons between primate **(A,C)** and rodent visual systems **(B,D)**. Schematic diagrams in **(A,B)** illustrate the ocular structures and fiber tracts originating from either left (red) or right (blue) visual cortex. Labeled structures include the retina, optic nerve (ON), optic chiasm (OC), optic tract (OT), superior colliculus (SC), lateral geniculate nucleus (LGN), and visual cortex (VC). In primates, around 53% of ON fibers cross the optic chiasm and project to the contralateral hemisphere (Kupfer et al., 1967). In contrast, more than 90% of rodent ON fibers project to the contralateral hemisphere after reaching the optic chiasm, while the remaining 5–10% of fibers project to ipsilateral hemisphere (Forrester and Peters, 1967). **(C,D)** are the corresponding Mn-enhanced MRI (MEMRI) scans after unilateral intravitreal injection into a marmoset and a rat respectively. **(C)** is in 2D oblique view, whereas **(D)** is in 3D axial maximum intensity projected view. Note the unilateral enhancement in the eye and the ON for both primate and rodent MEMRI. In the OT, LGN and SC, primate MEMRI showed bilateral enhancement as compared to unilateral enhancement in rodent MEMRI in the contralateral hemisphere of the injected eye. **(C,D)** are reproduced with permissions from Yamada et al. (2008) and Chan et al. (2017).

The visual system is a vital and complex component of the central nervous system that receives and processes electrochemical information for visual perception. Not only does it comprise the specialized sensory organ (i.e., the eye), but also the optic nerve fibers, the visual brain nuclei, as well as the feedforward and feedback pathways to and from the visual cortex (Gilbert and Li, 2013) (Figure 1A,B). To understand the mechanisms of visual perception in health and disease, it is imperative to focus not only on the anatomy and physiology of the eye itself, but also the visual brain connections as well as their interactions in different types of visual impairments in an objective, quantitative, and non-invasive manner.

Magnetic resonance imaging (MRI) offers non-destructive probing of the structure and function of the neural circuits without depth limitation. It can also help identify abnormalities in brain tissues *in vivo*. Among the advanced MRI techniques, manganese-enhanced MRI (MEMRI) has substantiated in the past two decades as a valuable tool for visualizing the architecture and physiology of the brain and the peripheral structures. MEMRI has a number of advantages over existing MRI techniques including blood-oxygen-level-dependent functional MRI (BOLD fMRI), diffusion tensor imaging, and other contrast-enhanced MRI systems. In its ionic form (Mn^2+^), manganese is paramagnetic, which makes it a suitable contrast agent that positively enhances T1-weighted MRI intensities in local tissues as a result of contrast uptake over a defined period of time. Manganese (II) chloride (MnCl_2_) is the most commonly used exogenous salt in the delivery of Mn^2+^ to the central nervous system *in vivo*. MEMRI is useful in basic and preclinical neuroscience because Mn^2+^ can be actively transported along axons and can accumulate in brain tissues with respect to the level of connectivity and activity, thereby aiding in enhancing neuroarchitecture contrast and in functional brain mapping (Pautler et al., 1998; Silva and Bock, 2008; Massaad and Pautler, 2011; Chan et al., 2014a) (Figure 1C,D). Mn^2+^ administration and transport can be performed when the animals are awake, thus the effects of anesthesia on neuronal activity and Mn^2+^ transport can be avoided or minimized. Together with the availability of fast sequences for T1-weighted imaging and longitudinal relaxivity (R1) mapping (Chuang and Koretsky, 2006; Tambalo et al., 2009), MEMRI can be performed *in vivo* in studies with longitudinal design, though a caveat of toxicity at higher Mn^2+^ doses does exist. With whole brain imaging and temporal assessments, the same tissues or different tissues of the same animals can serve as an internal control of the experimental brain targets at baseline and over time. Due to its ability to enhance neuronal tracts, detect activity-dependent changes, and reveal changes in axon integrity, MEMRI is an excellent tool for analyzing neuronal structures within the visual system and for providing insights into the mechanisms underlying neurodegenerative conditions of the visual system (e.g., glaucoma, diabetic retinopathy, and retinitis pigmentosa) as well as neuroplasticity, neuroprotection, and neuroregeneration (Liang et al., 2011; Haenold et al., 2012; Sandvig and Sandvig, 2014; Van der Merwe et al., 2016). In this review, we aim to provide an overview of the use of MEMRI for studying ophthalmology and visual neuroscience. We will discuss the underlying mechanisms of Mn^2+^ transport and accumulation in the eye and the brain, the routes of administrations, chemical and imaging preparation, and the metabolism and safety of Mn^2+^ based contrast. We will focus on four major domains of scientific inquiry where MEMRI can be put to imperative use — deciphering neuroarchitecture, tracing neuronal tracts, detecting neuronal activity, and identifying or differentiating glial activity. Additionally, we will appraise the possible limitations of this modality and consider new opportunities for further investigations.

## Mechanisms of Mn^2+^ Transport and Accumulation *In Vivo*

Due to its similarities with Ca^2+^ in terms of atomic size, chemistry, and valence, divalent manganese ions (Mn^2+^) tend to act as a Ca^2+^ analog by passing through voltage-gated calcium channels, making MEMRI an effective technique for visualizing neural activity *in vivo*. To bolster this hypothesis, mice deficient in L-type Ca^2+^ channel 1.2 (Ca_v_1.2) were found to have a near 50% reduction in signal enhancement in MEMRI compared to control mice (Bedenk et al., 2018). These results demonstrate that (a) Ca_v_1.2 is an important gateway for Mn^2+^ ions into neurons, and (b) Mn^2+^ biochemistry in neuronal milieu is similar to that of Ca^2+^ (except for their magnetic properties). Due to its unpaired electrons, Mn^2+^ is paramagnetic and acts as a positive contrast agent with short T1 relaxation time. As a result, tissues with high concentrations of Mn^2+^ generally appear as regions with bright signals in T1-weighted images.

Mn^2+^ ions are anterograde tract tracers, traveling away from the soma and toward projection terminals of neurons, then crossing the synapses before entering neighboring neurons. They can localize into the endoplasmic reticulum or Golgi bodies, and be actively transported along axons (Pautler, 2004; Watanabe et al., 2004; Van der Linden et al., 2007). The exact mechanism of Mn^2+^ transport is still elusive, but there is evidence to support a microtubule-dependent fast axonal transport mechanism. One study in Wistar rats involved unilateral administration of colchicine, a mitotic poison that inhibits microtubule polymerization, followed by bilateral administration of Mn^2+^ into the substantia nigra (Sloot and Gramsbergen, 1994). Axonal Mn^2+^ transport from the substantia nigra to the striatum was significantly decreased 48 h after Mn^2+^ injection in the colchicine-injected site compared to the control, suggesting that Mn^2+^ transport relies on microtubule polymerization (Sloot and Gramsbergen, 1994). Similar findings were also observed with unilateral injection of colchicine into the rat vitreous, which led to decreased Mn^2+^ enhancement in the ipsilateral optic nerve and contralateral superior colliculus (Hernandez et al., 2015).

The unique properties of Mn^2+^ gives MEMRI a number of advantages over a variety of contrast agents, BOLD fMRI, and diffusion tensor imaging. Compared to the passive T1 contrast agents such as gadolinium compounds (Chan et al., 2008b, 2012b; Ho et al., 2014), Mn^2+^ is an active contrast agent that can be used to detect activity-dependent changes and trans-synaptic transport *in vivo*. Since Mn^2+^ transport is independent of hemodynamic influences, MEMRI avoids the shortcomings seen in BOLD fMRI such as artifacts from draining veins and potential mismatches between neuronal activation and hemodynamic changes (Lin and Koretsky, 1997; Duong et al., 2000). Additionally, the use of Mn^2+^ as a direct tracer allows it to resolve fiber directions in areas of high curvature or tract crossing more easily than fiber modeling with diffusion tensor imaging (Lin et al., 2001). Mn^2+^ may reveal specifics of intracellular function (Barandov et al., 2019) while diffusion MRI may reflect transports in both intra- and extra-axonal spaces (Cheung et al., 2009; Hori et al., 2012; Wang Y. et al., 2015; Chung et al., 2018), though decisive opinions in the light of present literature are uncertain. It is possible to combine diffusion tensor imaging and MEMRI by using the same spatial parameters when performing both scans and overlaying the images (Lin et al., 2001). Diffusion tensor imaging has been verified by MEMRI in Mn^2+^-enhanced optic tracts and frontal eye fields (Lin et al., 2001; Yamada et al., 2008; Schaeffer et al., 2018) and vice versa across species. MEMRI is non-invasive which allows for effective longitudinal studies on brain structures — an advantage over histological studies which require sacrificing the animals. Diffusion tensor imaging and MEMRI can also complement one another to elucidate connectivity changes along the visual pathway in different optic neuropathy models (Thuen et al., 2009; Ho et al., 2015; Kancherla et al., 2016; Yang et al., 2018). Accumulation of Mn^2+^ ions in cells and neuronal tracts appears to be a proportionate marker of the neuronal activity and axonal integrity, making it valuable for probing both structural and functional connectivity between proximal and distal ends of the neural circuits *in vivo*.

## Routes of Administration

**FIGURE 2:**
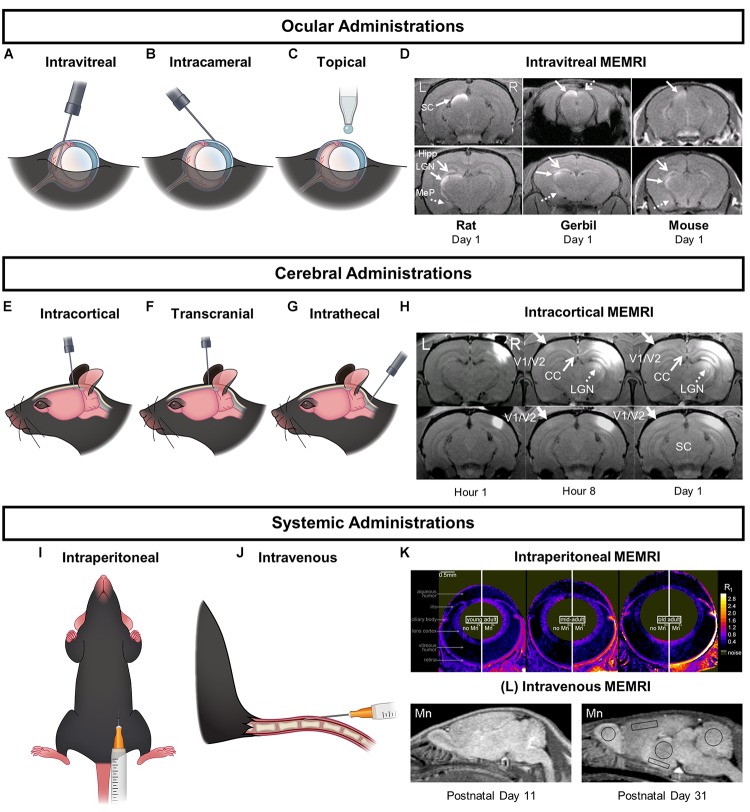
Major Mn^2+^ delivery routes and the corresponding MEMRI enhancement patterns in the visual system. **(A–C,E–G,I**,**J)** are schematic representations of ocular, cerebral, and systemic injection routes respectively. **(D,H,K)** are the corresponding MEMRI enhancement patterns in the brain as a result of the specific Mn^2+^ administrations. **(D)** is a series of MEMRI scans of the rat, gerbil, and mouse brains 1 day after an intravitreal Mn^2+^ injection. Mn^2+^ enhancement could be found in the contralateral SC and LGN along the central visual pathway, and in the non-visual regions in the contralateral hippocampus (Hipp) and medial posterior amygdala (MeP). **(H)** contains MEMRI scans of a rat brain after intracortical Mn^2+^ injection to the right V1/V2 transition zone at 1-, 8-, and 24-h time points. Mn^2+^ enhancement could be observed along the cortico-cortical pathways in the left contralateral V1/V2 border and the splenium of corpus callosum. Mn^2+^ was also seen to transport along the cortico-subcortical feedback pathways in the ipsilateral LGN and SC. MEMRI scans in **(K)** portray age-related increase in outer retinal Mn^2+^ uptake in Long Evans rats between 2.4 and 19 months old. MEMRI was taken at baseline and at about 4 h after intraperitoneal Mn^2+^ administration using quantitative mapping of tissue R1 values in units of s^-1^. **(L)** shows sagittal MEMRI scans of postnatal days (PD) 11 and 31 rats at 24 h after intravenous Mn^2+^ injection. Brain Mn^2+^ uptake appeared higher in neonates and decreased with brain development. An estimate of the relative cortical concentration of manganese uptake shows a twofold drop from PD 11 to PD 31. **(D,H,K,L)** are reproduced with permissions from de Sousa et al. (2007), Bissig et al. (2013), and Chan et al. (2014a, 2017).

Mn^2+^ can be delivered to the brain regions of interest via local intraocular and intracerebral administrations or systemic administrations into the blood stream (Figure 2). When choosing an appropriate delivery route, it is essential to consider the purpose of Mn^2+^ detection, the species, age, size, and gender of animal models, the frequency and dosage of Mn^2+^ administration, as well as the expected outcomes and caveats of each administration. These considerations are important as they may reflect different sensitivity, specificity, and mechanisms of Mn^2+^ enhancement in the brain targets (Silva et al., 2004; Silva and Bock, 2008; Chan et al., 2017). Furthermore, Mn^2+^ ions are toxic at high concentrations and may damage the target tissues or the local sites of administration (Bock et al., 2008; Thuen et al., 2008; Lin et al., 2014a; Vousden et al., 2018). It is pertinent to consider the cumulative effects of Mn^2+^ toxicity and design careful experiments especially if longitudinal assessments, functional/behavioral outcomes or histological studies are needed after Mn^2+^ injection. In most *in vivo* studies, Mn^2+^ is delivered as a bolus injection of MnCl_2_ solution. If a large amount of Mn^2+^ needs to be administered, continuous low-dose release or fractionation may minimize toxicity and hence bring down the probability of the adverse effects (Bock et al., 2008, 2009; Grunecker et al., 2010; Mok et al., 2012; Morch et al., 2012; Driencourt et al., 2017; Poole et al., 2017; Vousden et al., 2018). In the following paragraphs, we will elucidate a number of delivery routes used in MEMRI experiments of the visual system as well as relevant precautions of their use.

Intravitreal MnCl_2_ injection is commonly used to investigate neuronal tracts of the central visual pathway by visualizing the retina, optic nerve, optic chiasm, superior colliculus, lateral geniculate nucleus, and sometimes the visual cortex (Watanabe et al., 2001; Murayama et al., 2006; Chan et al., 2008c, 2014a; Thuen et al., 2008; Yamada et al., 2008; Lindsey et al., 2013) at fractionated doses or at extended time points in T1-weighted images. It can also enhance the nearby non-visual brain regions such as hippocampus and amygdala (Chan et al., 2017). High concentrations of MnCl_2_ risk damaging the retinal ganglion cells, as well as compromising the integrity of other ocular structures such as the corneal stroma, endothelium, anterior chamber, lens, and outer retina (Thuen et al., 2008; Haenold et al., 2012; Luo et al., 2012; Xiao et al., 2019).

Topical administration of MnCl_2_ solution as eye drops onto the corneal surface presents a non-invasive and simple technique that can deliver Mn^2+^ into the visual system. While topical Mn^2+^ loading can enhance the anterior chamber, cornea, iris, retina, and the posterior visual pathways in the lateral geniculate nucleus and superior colliculus (Sun et al., 2012), no apparent enhancement in the vitreous chamber has been reported through this method, indicating that transportation of Mn^2+^ into the central visual pathway may not involve the vitreous and is likely independent of transcorneal diffusion (Sun et al., 2011; Lin et al., 2014a; Liang et al., 2015). Instead, uptake may involve permeation across the conjunctiva and sclera into the anterior uvea (Sun et al., 2011). Mn^2+^ uptake via this route can be significantly improved by surgical removal of the corneal epithelium, which indicates the presence of the physical corneal barrier to topical Mn^2+^ administration (Chen et al., 2016). To improve non-invasive Mn^2+^ entry into the eye, Mn^2+^ ions can be administered via transscleral and transcorneal iontophoresis by application of local electric current (Li et al., 2004). Transscleral iontophoresis leads to enhancements in the vitreous while transcorneal iontophoresis leads to Mn^2+^ filling of the anterior chamber (Li et al., 2004).

Intracameral MnCl_2_ injection has also been used to enhance the retina, optic nerve, superior colliculus, lateral geniculate nucleus, and visual cortex (Lindsey et al., 2007, 2013). However, this technique is less common likely because it is invasive while it does not provide substantial advantages over the topical loading. Additionally, it is not as direct as intravitreal injection for Mn^2+^ deposition into the retina.

Intracortical injection of MnCl_2_ to the rodent visual cortex has also been performed which results in Mn^2+^ enhancement in the splenium of corpus callosum, contralateral V1/V2 border, and the ipsilateral dorsal lateral geniculate nucleus and superior colliculus along the feedback pathway (Chan et al., 2012a). Recently, intracortical MnCl_2_ injection into the frontal eye field of primates has been used to study the neural circuits connected to this region (Schaeffer et al., 2018). These brain regions are areas that intraocular or topical administration cannot easily reach (Watanabe et al., 2001; Thuen et al., 2008).

Intrathecal or intracerebroventricular injection is another important route of Mn^2+^ administration that allows Mn^2+^ delivery to brain tissues while circumventing the blood–brain barrier. This technique requires a high level of expertise and precision comparable to intracortical injection. In a study carried out on Sprague-Dawley rats, MnCl_2_ injections through the cisterna magna resulted in Mn^2+^ enhancement around the cerebrospinal fluid space in the olfactory bulb, cortex, and the brain stem within the first 6 h of administration which lasted for at least 3 weeks (Liu et al., 2004). Mn^2+^ uptake from the cerebrospinal fluid circulation into neuronal structures likely includes mechanisms apart from passive diffusion (Bock et al., 2008, 2009), such as glia-mediated cerebrospinal fluid-interstitial fluid exchange (Iliff et al., 2013), though further investigations are needed to confirm the specificity of Mn^2+^ enhancement via this pathway. Alternatively, intranasal administration of MnCl_2_ can bypass the blood–brain barrier while enhancing the visual cortex (Fa et al., 2010). It is also possible to assess the neuronal tracts of the visual system directly by transcranial Mn^2+^ delivery (Atanasijevic et al., 2017).

Systemic administrations such as intraperitoneal and intravenous Mn^2+^ injections have also been used to visualize the retina (Berkowitz et al., 2006; Braun et al., 2007; Bissig and Berkowitz, 2011), primary visual cortex (Bissig and Berkowitz, 2009), and other subcortical visual structures. Mn^2+^ transport in the brain via this route appears to stem from the blood supply to the pituitary gland, which then enters the cerebrospinal fluid through the choroid plexus, and then into brain regions near the ventricles, such as the striatum, thalamus, cerebellum, and hippocampus (Aoki et al., 2004; Bock et al., 2008, 2009; Alaverdashvili et al., 2017). However, the blood–brain and blood–retinal barriers pose a challenge when it comes to standardizing doses of systemic Mn^2+^ administration to maximize bioavailability and neuroenhancement while avoiding systemic toxicity. Hyperosmolar agents can be used to temporarily breach the blood–brain barrier to improve Mn^2+^ entry in the visual cortex and other brain regions after systemic Mn^2+^ infusion (Aoki et al., 2002; Fa et al., 2011). Alternatively, low MnCl_2_ concentrations have been successfully administered intravenously in fractionated doses to enhance the primary visual cortex in marmosets (Bock et al., 2009), as well as the periventricular areas in rodents without compromising the blood–brain barrier (Talley Watts et al., 2015; Alaverdashvili et al., 2017). Subcutaneous Mn^2+^ injection or infusion may also allow slow release of Mn^2+^ into the bloodstream, thus avoiding immediate hepatic elimination resulting in prolonged Mn^2+^ accumulation in the brain compared to intravenous administration (Chuang et al., 2009; Shazeeb and Sotak, 2012; Vousden et al., 2018).

Oral administration of MnCl_2_ has recently been used to reveal differences in brain development between male and female neonates (Qiu et al., 2018), though oral Mn^2+^ administration for studying adult rodent visual system is currently a less preferred method given the lower bioavailability as compared to intraperitoneal and intrathecal routes (Roels et al., 1997).

## Contrast Agent Preparation and MRI Protocols

Appropriate choices of the buffer system, concentration, and pH are indispensable to the success of MEMRI experiments. MnCl_2_ is available in different grades of purification (mostly > 99% purity) and various anhydrous and hydrated forms. Since mammalian body fluids have around 300 mOsm/L of osmolarity (Bhave and Neilson, 2011; Fregoneze et al., 2014; Hooper et al., 2015; Villiger et al., 2018), the infused MnCl_2_ solution should be prepared at similar osmotic concentrations, especially for local injections. While the buffer solution of MnCl_2_ can be deionized water, saline, phosphate-buffered saline, etc., physiological pH of approximately 7.2–7.4 should be maintained as lower pH may cause acidosis of the target tissues while higher pH may cause alkalosis. For example, the pH of a 300 mOsm/l MnCl_2_ solution prepared from deionized water is 5.5 to 5.8 under standard temperature and pressure conditions (Silva et al., 2004). Bicine and sodium hydroxide can be utilized to adjust pH to around 7.2 to 7.4 (Silva et al., 2004; Bock et al., 2008). Alternatively, for non-invasive Mn^2+^ delivery, such as transcranial administration, high osmolarity of MnCl_2_ solution at about 250–500 mM is required to pass through the intact rat skull unless CaCl_2_ is added to MnCl_2_ such that the total salt concentration equals 500 mM (Atanasijevic et al., 2017). It should be noted that retinal degeneration has been reported after intravitreal injection of normal saline but not phosphate-buffered saline to C57BL/6J mice (Hombrebueno et al., 2014). Caution is warranted when determining the dosage and chemical property of the buffer solution for localized MnCl_2_ injection.

On the other hand, the intensity of T1-weighted imaging is dependent on the gradient, the radiofrequency field homogeneity, and the coil sensitivity of the MRI system, which often differ slightly between experimental sessions. To account for such inhomogeneity, a small phantom filled with saline, water, or MnCl_2_ solution can be placed near the animal’s head to normalize the neural tissue signals to the phantom signals in the same imaging slices during acquisition (Chan et al., 2008c, 2017; Yang et al., 2018). Alternatively, the contralateral homotopic tissues or the nearby muscles may be used as the internal control for the eye or the brain after local but not systemic Mn^2+^ administration (Thuen et al., 2005), assuming negligible T1-weighted signal enhancement in these regions soon after local injection. Signal intensities may also be normalized to the background noise from each animal (Aoki et al., 2004; Thuen et al., 2005). One may also use T1-weighted imaging sequences that are less sensitive to radiofrequency inhomogeneity (Lee et al., 1995; Thomas et al., 2005). For more accurate quantitation, R1 mapping should be performed to compute the absolute R1 parametric values of the target tissues which are much less affected by signal non-uniformity than the relative signal comparisons in T1-weighted imaging. There are a few fast sequences available for R1 mapping (Chuang and Koretsky, 2006; Tambalo et al., 2009), whereas R1 is noted to linearly correlate with tissue Mn^2+^ concentration in Mn^2+^-injected animals (Chuang et al., 2009).

## Neuroarchitecture Evaluation

Manganese-enhanced MRI of neuroarchitecture evaluation involves utilizing Mn^2+^ administration to detect the cytoarchitecture of brain tissues. The underlying principle of this technique is based on the varying degrees of Mn^2+^ accumulation resulting from differences in tissue properties at basal levels such as Mn^2+^ uptake through activity-dependent Ca_v_1.2 channels in active neurons (Bedenk et al., 2018). A variety of neural circuits can be enhanced through systemic or local delivery of Mn^2+^. The high sensitivity and resolution provided by Mn^2+^ distribution allows for *in vivo* brain mapping, as well as outlining of retinal and cortical layers which, in turn, can be used to differentiate between disease conditions to discern neuropathological mechanisms. The specificity and accuracy of Mn^2+^ enhancements have been determined via histological confirmation. A list of relevant MEMRI studies can be found in Table 1. In this section, we will go through few important studies that have implemented the use of Mn^2+^ to resolve the neuroarchitecture of the visual system in high-resolution MRI.

**Table 1:** Summary of MEMRI protocols used for the detection of neuroarchitecture in the visual system in terms of species, delivery route, Mn^2+^ dose, magnetic field strength and anatomical structures enhanced and studied.

Species	Delivery route	Mn^2+^ dose	Field strength	Anatomical structures of interest						Citation
					
				Retina	ON	SC	LGN	VC	Others	
FVB mice	Intravenous	88 mg/kg; 120 mM; 250 μL/h	11.7 T					✓		Lee et al., 2005
C57BL/6J mice	Subcutaneous osmotic pump	180 mg/kg; 0.25–1.0 μL/h	7 T					✓		Mok et al., 2012
RCS rats, Sprague-Dawley rats	Intravitreal	5 μL; 30 mM	4.7 T	✓						Nair et al., 2011
Sprague-Dawley rats	Intrathecal	50 μL; 25 mM	4.7 T					✓		Liu et al., 2004
	Intravenous	2.0 mL; 64 mM; 1.8 mL/h	11.7 T				✓	✓		Aoki et al., 2004
	Subcutaneous	75, 150, 300 mg/kg; 25, 50, 100 mM	2 T					✓		Shazeeb and Sotak, 2012
	Intraperitoneal	45 mg/kg; 100 mM	7 T			✓		✓		Chan et al., 2017
Old-World fruit bats	Intraperitoneal	3 mL/kg	7 T			✓				Liu et al., 2015
Common marmosets	Intravenous	40 mM; 1.25 mL/h	7 T					✓		Bock et al., 2009


*If a study examined dose-dependent effects, the reported optimal dose was chosen (ON, optic nerve; SC, superior colliculus; LGN, lateral geniculate nucleus; VC, visual cortex).*

**FIGURE 3:**
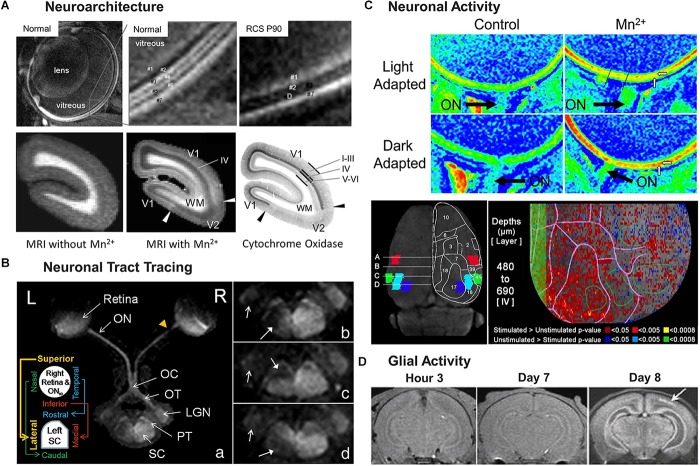
Illustrations of four key MEMRI applications for studying the visual system, from neuroarchitecture detection **(A)**, to neuronal tract tracing **(B)**, neuronal activity detection **(C)** and glial activity identification **(D)**. **(A)** represents detection of neuroarchitecture in the rodent retina and the primate visual cortex. Top row of **(A)** shows MEMRI detection of distinct bands of the normal (left and middle) and degenerated rodent retinas (right) with alternating dark and light intensity signals, as denoted by the numbering of layers. Note the compromised photoreceptor layer “D” upon degeneration in the Royal College of Surgeons (RCS) rats at postnatal day (P) 90. Bottom row of **(A)** represents *in vivo* T1-weighted MRI of the marmoset occipital cortex before (left) and after (middle) systemic Mn^2+^ administration. The corresponding histological section stained for cytochrome oxidase activity is shown on the right. The arrows indicate the primary/secondary visual cortex (V1/V2) border; I–III, IV, and V–VI indicate the cortical layers; and WM represents white matter. V1 detected in the T1-enhanced MEMRI scans agrees with the V1 identified in the histological section. The cortical layer IV experiences the strongest layer-specific enhancement, defining the extent of V1. **(B)** represents the use of MEMRI tract tracing for retinotopic mapping of normal and injured central visual pathways in Sprague-Dawley rats. MEMRI was performed 1 week after partial transection to the right superior intraorbital optic nerve (ON_io_) in **a,b** as shown by the yellow arrowhead in **a**, and to the temporal and nasal regions of the right optic nerve in **c,d**, respectively. After intravitreal Mn^2+^ injection into both eyes, the intact central visual pathway projected from the left eye could be traced from the left retina to the left optic nerve (ON), optic chiasm (OC), right optic tract (OT), right lateral geniculate nucleus (LGN), right pretectum (PT), and right superior colliculus (SC) in **a**. In contrast, reduced anterograde Mn^2+^ transport was found beyond the site of partial transection in the central visual pathway projected from the right eye in a retinotopic manner following the schematics in the insert in **a**. **b–d** in the right column highlight the reduced Mn^2+^ enhancement in the lateral, rostral and caudal regions of the left SC, denoted by the solid arrows. Open arrows indicate the hypointensity in the left LGN. **(C)** shows the use of MEMRI for detection of neuronal activity in the retina (top 2 rows) and the visual cortex (bottom row) of rodents. The heat maps on the top 2 rows of **(C)** visualize retinal adaptation by MEMRI in either light or dark condition. The horizontal white arrows mark the enhanced inner retina 4 h after systemic Mn^2+^ administration (right column) as compared to the control condition without Mn^2+^ administration (left column), while the vertical white arrows point to the outer retina that has higher intensity in dark-adapted than light-adapted conditions. The optic nerve (ON) is identified by a black arrow in each image. The bottom row of **(C)** represents neuronal activity of the visual cortex after systemic Mn^2+^ administration and awake visual stimulation. The left image shows the anatomy of cortical regions of interest (ROIs) in terms of Brodmann areas: blue for the binocular division of the primary visual cortex (Area 17), cyan for the lateral division of the accessory visual cortex (Area 18), red for the primary somatosensory cortex (Area 2), and green for the primary auditory cortex (Area 41). A superimposed drawing shows the relevant surface topography. On the right is a voxel-wise analysis of activity-dependent Mn^2+^ enhancement in one hemisphere centered in layer IV of the primary visual cortex at a depth from 480 to 690 μm. The top of the image is the rostral side of the cortex while the left side depicts the position of the longitudinal fissure. Values of the P-threshold are indicated on the bottom. The primary visual cortex, represented by the leftmost green open circle, had the highest density of below-threshold voxels. The green shaded band to the left, centered at the longitudinal fissure, is a buffer of the unanalyzed space. **(D)** shows a series of T1-weighted images of neonatal rats at 3 h, and 7 and 8 days after mild hypoxic-ischemia (H-I) insult at postnatal day (P) 7. The injury was induced by unilateral carotid artery occlusion and exposure to hypoxia at 35°C for 1 h. After MRI scans at day 7, systemic Mn^2+^ administration was performed, and the image at day 8 represents MEMRI enhancement. The white arrow points to gray matter injuries in the ipsilesional hemisphere around the visual cortex. This type of gray matter lesion is not visible in the images from hour 3 and day 7 post-insult. Immunohistology of the same rats suggested co-localization of overexpressed glial activity in the same lesion area in MEMRI (not shown). **(A–D)** are reproduced with permissions from Berkowitz et al. (2006), Yang and Wu (2007), Bissig and Berkowitz (2009); Bock et al. (2009), Chan et al. (2011), and Nair et al. (2011).

Manganese-enhanced MRI enables *in vivo* visualization of anatomical details in the whole brain from topographical and functional domains to layer-specific or even cellular levels (Aoki et al., 2004; Lee et al., 2005; Bock et al., 2009; Chuang et al., 2010; Chan et al., 2014a). With regards to the visual system, MEMRI has been used to reveal retinal and cortical structures layer-specifically in healthy and diseased animal models. For instance, after intravitreal MnCl_2_ injection into normal rats, MEMRI at 25 micron resolution displayed seven bands of alternating high and low intensities (Figure 3A) which correspond to the ganglion cell layer, inner plexiform layer, inner nuclear layer, outer plexiform layer, outer nuclear layer, photoreceptor segment layer, and choroidal vascular layer (Nair et al., 2011). In contrast, the Long-Evans Royal College of Surgeons (RCS) rats with inherited photoreceptor degeneration only showed four bands of alternating intensities alongside with one debris band (Nair et al., 2011) (Figure 3A) indicating loss of the cellular architecture in the retina. Histological analysis confirmed the findings revealing four intact retinal layers and a debris layer in place of the outer plexiform layer, outer nuclear layer, and photoreceptor segment layer.

The architecture of the visual cortex can also be visualized using MEMRI. For instance, fractionated intravenous MnCl_2_ injections to marmosets led to significantly greater enhancement in the V1 and V2 gray matter regions of the visual cortex compared to the proximal white matter in MEMRI, with V1 being slightly more enhanced than V2 (Bock et al., 2009) (Figure 3A). The cortical layer IV in V1 experienced the strongest layer-specific enhancement. These differences in signal intensities allowed investigators to identify the borders between corresponding brain structures in the images. Cytochrome oxidase staining of brain samples corroborated with MEMRI observations, supporting the use of high-resolution MEMRI in resolving cytoarchitecture (Bock et al., 2009).

## Neuronal Tract Tracing

Early methods of neuronal tract tracing involve the use of biotinylated dextran (Rajakumar et al., 1993), horseradish peroxidase (Kristensson and Olsson, 1971), fluorogold (Tillet et al., 1993), herpes simplex virus (Sun et al., 1996), and carbocyanine fast DiI (Friedland et al., 1996). However, these methods are mostly mono-synaptic and require sacrificing the animals to obtain brain slices for visualizing the tracts. In addition, the process of serial brain sectioning for reconstructing three-dimensional information is laborious, requires highly skilled expertise and is prone to errors. For this reason, it needs a more efficient tract tracing method that is viable for living animals, and MEMRI serves this purpose. Since Mn^2+^ can be readily taken up and transported by neurons, it can be used as an *in vivo* anterograde tracer for MRI to determine the movement and distribution of this moiety which, in consequence, allows tracing of the neuronal tracts. In order to examine the veracity of this premise, one of the earliest MEMRI experiments involved administration of MnCl_2_ solution into the vitreous to unveil the anatomical structures along the central visual pathway (Pautler et al., 1998). The rate of anterograde transport of Mn^2+^ was found to be about 2–8 mm/h in rodents (Sandvig et al., 2011), and is dependent upon body temperature (Smith et al., 2007), age (Minoshima and Cross, 2008; Chan et al., 2012a), and health status (Gallagher et al., 2012; Lin et al., 2014b; Majid et al., 2014; Bertrand et al., 2018). In addition, trans-synaptic transport of Mn^2+^ to the distal neurons in the visual cortex can be detected upon sufficient functional activity (Bearer et al., 2007; Lindsey et al., 2007; Chan et al., 2014a). These results demonstrate that it is plausible to use Mn^2+^ to trace separate tracts simultaneously in the same animals and to examine the physiological transport properties in chronic studies. Further investigations are needed to (a) determine the sensitivity and specificity of MEMRI for neuronal tract tracing; (b) determine the doses at which Mn^2+^ becomes toxic; (c) clarify the mechanism of how Mn^2+^ enters the cells, and (d) decipher the mechanism through which Mn^2+^ is transported across the cellular milieu. In this section, we will examine important studies that utilize MEMRI to trace neuronal tracts in the visual system. A summary of the relevant MEMRI protocols used is provided in Table 2.

**Table 2:** Summary of MEMRI protocols used for neuronal tract tracing in the visual system in terms of species, delivery route, Mn^2+^ dose, magnetic field strength and anatomical structures enhanced and studied.

Species	Delivery route	Mn^2+^ dose	Field strength	Anatomical structures of interest						Citation
					
				Retina	ON	SC	LGN	VC	Others	
C57BL/6J mice	Topical	5 μL; 1–1.5 M	4.7 T	✓	✓	✓	✓			Sun et al., 2011
	Intravitreal	2 μL; 1 M		✓	✓	✓	✓			
		0.5 μL; 100 mM	9.4 T		✓	✓	✓			Ho et al., 2015
C57BL/6J mice, DBA/2J mice	Intravitreal	1 μL; 100 mM	7 T		✓	✓	✓			Fiedorowicz et al., 2018
		0.5 μL; 100 mM	9.4 T		✓	✓	✓			Yang et al., 2018
C57BL/6J mice, EAE mice	Intravitreal	0.25 μL; 200 mM	4.7 T		✓					Lin et al., 2014b
C57BL/6J mice, APP-/- mice	Intravitreal	0.25 μL; 200 mM	11.7 T			✓	✓			Gallagher et al., 2012
C57BL/6J mice, CBA mice, KCL1-/- mice	Intravitreal	0.25 μL; 200 mM	11.7 T		✓	✓	✓			Bearer et al., 2007
C57BL/6J mice, NF-κB p50^KO^ mice	Intravitreal	2 μL; 7.5 mM	3 T	✓	✓	✓	✓			Fischer et al., 2014
NIH-Swiss white mice	Intracameral	1 μL; 1 M	7 T			✓	✓	✓		Lindsey et al., 2007
FVB mice	Intravitreal	1–2 μL; 800 mM	7 T	✓	✓	✓				Pautler et al., 1998
129Sv/J mice	Intravitreal	2 μL; 20 mg/mL	7 T		✓					Mansergh et al., 2014
C57BL/6J mice, Fischer rats, frogs, fish	Intravitreal	3 μL; 50 mM	2.35 T		✓					Sandvig et al., 2011
Mongolian gerbils	Intravitreal	2 μL; 100 mM	7 T	✓	✓	✓	✓			Chan et al., 2017
Wistar rats	Intravitreal	0.1 μL; 1 M	2.35 T	✓	✓	✓	✓			Watanabe et al., 2001
Fischer rats	Intravitreal	3 μL; 50 mM	2.35 T	✓	✓	✓				Thuen et al., 2005
		4 μL; 3.9 M	7 T		✓					Ryu et al., 2002
Sprague-Dawley rats	Intravitreal	3 μL; 50 mM, or 2 μL; 100 mM	7 T	✓	✓	✓	✓		Hipp, Amy	Chan et al., 2008c, 2011, 2017
		4 μL; 200 μM	7 T		✓	✓				Hernandez et al., 2015
		3 μL; 30 mM	1.5 T	✓	✓	✓	✓			Yang et al., 2016
		2 μL; 200 mM	3 T		✓	✓	✓			Tang et al., 2017a
		1.5 μL; 100 mM	9.4 T		✓	✓	✓			van der Merwe et al., 2019
	Intravitreal	3 μL; 50 mM (x3)	7 T		✓	✓	✓	✓		Chan et al., 2014a
	Subcortical	30 nL; 100 mM					✓	✓		
	Intracortical	100 nL; 100 mM				✓	✓	✓	CC	
	Transcranial	50 μL; 10–500 mM	11.7 T			✓	✓	✓		Atanasijevic et al., 2017
	Inner ear perilymph	6 μL; 200 mM	3 T			✓	✓	✓		Tang et al., 2017b
Syrian golden hamsters	Intravitreal	2 μL; 200 mM	7 T		✓	✓	✓			Liang et al., 2011
Old-World fruit bats	Intravitreal	2 μL; 120 mM	7 T	✓	✓	✓	✓	✓		Liu et al., 2015
Common marmosets	Intravitreal	0.5 μL; 1 M	7 T	✓	✓	✓	✓			Yamada et al., 2008
Rhesus macaques	Intravitreal	75–100 μL; 1–1.5 M	4.7 T	✓	✓	✓	✓	✓	ITC	Murayama et al., 2006
	Intracortical	5 μL;120 and 300 mM	7 T						FEF	Schaeffer et al., 2018
New Zealand rabbits	Topical	400 μL; 50–200 mM	3 T	✓	✓	✓				Chen et al., 2016
Pigmented rabbits	Intravitreal	25 μL; 5–40 mM	1.5 T		✓	✓	✓			Wang et al., 2016


*If a study examined dose-dependent effects, the reported optimal dose was chosen (ON, optic nerve; SC, superior colliculus; LGN, lateral geniculate nucleus; VC, visual cortex; FEFs, frontal eye fields; Hipp, hippocampus; Amy, amygdala; CC, corpus callosum; ITC, inferotemporal cortex).*

Watanabe et al. (2001) are among the earliest to perform extensive MEMRI studies to determine the spatiotemporal evolution of MEMRI for tracing the visual system in healthy rodents. At 8, 24, 48, and 72 h after intraocular MnCl_2_ administration to adult Wistar rats, the best MEMRI enhancement of the central visual pathway was achieved at 24 h post-injection, which revealed clear delineation of the retina, the axonal tracts, and the primary visual centers. At 8 h there was insufficient transport beyond the optic chiasm, while the contrast faded at 48 h. At 24 h, a continuous pattern of anterograde labeling was observed from the retina, optic nerve, and optic chiasm to the contralateral optic tract, the dorsal and ventral lateral geniculate nucleus, the superficial gray layer of the superior colliculus and its brachium, the olivary pretectal nucleus, and the suprachiasmatic nucleus. Enhancement was achieved on the ipsilateral hemisphere to a lesser degree after passing the optic chiasm, which agreed with the known anatomical projection in rodents (Forrester and Peters, 1967) (Figure 1). A shortcoming of MEMRI-based neuroanatomic tracing is its relatively low sensitivity to sparse fibers. Several tracts known to consist of only a few fiber connections could be visualized by other methods but were not distinguishable in this study. Some examples include the nuclei of the accessory optic system, lateral geniculate nucleus, and olivary pretectal nuclei in the same hemisphere of the rodent brain ipsilateral to the unilaterally injected eye.

While most MEMRI studies involving intraocular MnCl_2_ injection detected the retinal pathways toward the lateral geniculate nucleus or superior colliculus (Pautler et al., 1998; Watanabe et al., 2004; Thuen et al., 2005; Chan et al., 2011, 2017; Fischer et al., 2014; Xiao et al., 2019) (Figure 2D), detection of the visual cortex upon intraocular MnCl_2_ injection was less frequently reported (Murayama et al., 2006; Bearer et al., 2007; Lindsey et al., 2007; Chan et al., 2014a). The fact that Mn^2+^ is not easily visualized in the visual cortex appears to reflect differences in transport dynamics between the proximal and distal visual pathways. Along the rodent visual pathway, Mn^2+^ ions are taken up by the retinal ganglion cells, which experience anterograde axonal transport along the optic nerve and optic tracts, and subsequently accumulate at the axon terminals and the synapses at the contralateral superior colliculus and lateral geniculate nucleus (Pautler et al., 1998; Watanabe et al., 2004). At this point, the Mn^2+^ ions need to cross the synapse and be collected by the post-synaptic terminals of the next neurons in the distal pathway for further transport and signal enhancement. While *in vivo*, trans-synaptic tract-tracing has been reported across different nervous systems and species (Saleem et al., 2002; Pautler, 2004; Chan et al., 2014a; Almeida-Correa et al., 2018), the amount of transfer appears substantially smaller than the proximal Mn^2+^ input and is dependent upon the synaptic integrity, axonal caliber, and functional activity of the neurons (Murayama et al., 2006; Bearer et al., 2007). This may explain at least in part why trans-synaptic enhancement of the visual cortex by intraocular Mn^2+^ injection has been challenging and less sensitive to be detected (Lindsey et al., 2007). Since the degree of Mn^2+^ enhancement along the visual pathway depends on the duration of the available Mn^2+^ input in the eye more than the dosage used (Olsen et al., 2010), sustained ocular Mn^2+^ release and MEMRI at an extended time point might help improve the sensitivity of detecting visual cortex Mn^2+^ enhancement (Morch et al., 2012; Chan et al., 2014a).

Apart from the central visual pathway, MEMRI has been increasingly utilized as a robust tool for layer-specific and topographical brain mapping of the transcallosal, cortico-geniculate, cortico-collicular, poly-synaptic, and intracortical horizontal connections in the rodent visual system (Chan et al., 2012a, 2014a) (Figure 2H). In particular, the ability of MEMRI to appraise cortico-cortical and cortico-subcortical pathways opens up new avenues to explore the neurophysiological properties of top–down, feedback neural pathways to and from the visual cortex that have been largely understudied (Gilbert and Li, 2013). In addition to the rodent visual system, MEMRI is readily translatable to study the visual system of other species including primates. For example, intravitreal Mn^2+^ injection allows detection of not only the central visual pathways in marmosets (Yamada et al., 2008) (Figure 1C) and rhesus macaques (Murayama et al., 2006), but also higher levels of the ventral visual stream such as the inferotemporal cortex in rhesus macaques (Murayama et al., 2006). In the prefrontal cortex, a recent MEMRI study focused on the saccadic eye movement system of rhesus macaques, whose neural circuit was also documented histologically, thereby making it a useful reference point to verify MEMRI as a precise *in vivo* technique in analyzing long-range neuronal connectivity (Schaeffer et al., 2018). After intracortical Mn^2+^ injection into the frontal eye fields, comparisons between diffusion tensor imaging, MEMRI, and histochemical results revealed that the tract tracing via MEMRI was in agreement with histochemical tracing whereas diffusion tensor imaging underestimates connectivity (Schaeffer et al., 2018). The reason for this may be attributed to the limitation of diffusion tensor imaging to resolve crossing fibers. These results further support the use of MEMRI in investigating neuronal tracts and reveal a distinct benefit of this technique over diffusion tensor imaging.

Manganese-enhanced MRI also provides a solution to non-invasively image transport deficits in the visual system for examining neuronal abnormalities caused by optic neuropathies such as irradiation-induced injuries (Ryu et al., 2002), optic nerve crush (Thuen et al., 2005; Fischer et al., 2014), glaucoma (Chan et al., 2007, 2008c; Fiedorowicz et al., 2018; Yang et al., 2018), optic neuritis (Lin et al., 2014b), retinal ischemia/reperfusion (van der Merwe et al., 2019), and neonatal hypoxia-ischemia (Chan et al., 2014b). Studies by Thuen et al. (2005, 2009) are among the earliest to demonstrate the feasibility of MEMRI for longitudinal monitoring of Mn^2+^ transport along injured axons of the central visual pathway via intraocular Mn^2+^ administration. Accumulation of Mn^2+^ in the vitreous and enhancement plummeting at the site of injury in the optic nerve are commonly noted after neuronal damages due to blockade of Mn^2+^ transport (Thuen et al., 2005; Sandvig et al., 2012), whereas the extent of reduced Mn^2+^ enhancement at the distal site often reflects the severity of damages (Fischer et al., 2014; Lin et al., 2014b). Subsequent studies demonstrated the use of *in vivo* MEMRI tract tracing for retinotopic mapping at submillimeter resolution (Chan et al., 2011) (Figure 3B), and for monitoring primary versus secondary degeneration after partial transection of the optic nerve (Chan et al., 2017). With these premises, MEMRI can serve as an *in vivo* imaging model system to evaluate neuroprotective approaches to the injured visual system (Mansergh et al., 2014; Van der Merwe et al., 2016, van der Merwe et al., 2019), as well as to trace neuroplasticity (Immonen et al., 2008; Chan et al., 2012a; Tang et al., 2017b) and regenerated axons along the visual pathway (Liang et al., 2011; Sandvig et al., 2011, 2012; Sandvig and Sandvig, 2014; Komatsu et al., 2017). MEMRI can be combined with diffusion tensor imaging to give complementary information about injury and regeneration in the adult optic nerve (Thuen et al., 2009). In terms of correspondence between image modalities, our previous MEMRI and diffusion tensor imaging experiments indicated that anterograde Mn^2+^ transport is relatively more sensitive to axial diffusivity changes than radial diffusivity changes in the injured visual pathway (Ho et al., 2015).

## Evaluation of Neuronal Activity

Manganese-enhanced MRI of neuronal activity detection involves analyzing brain structures under a certain stimulus. Regions of the brain that are active in response to a stimulus experience a rise in ion demand, which results in increased Mn^2+^ accumulation after exogenous administration. Higher concentrations of Mn^2+^ ions in local brain regions result in higher T1-weighted signal intensities on MRI, indicating that signal enhancement of these structures can be a biomarker of their activity (Lin and Koretsky, 1997; Duong et al., 2000; Yu et al., 2005). In this section, we will overview findings which involved the use of MEMRI to examine neuronal activity in the visual system. A compilation of relevant studies can be found in Table 3.

**Table 3:** Summary of MEMRI protocols used for detecting neuronal activity in the visual system in terms of species, delivery route, Mn^2+^ dose, magnetic field strength and anatomical structures enhanced and studied.

Species	Delivery route	Mn^2+^ dose	Field strength	Anatomical structures of interest						Citation
					
				Retina	ON	SC	LGN	VC	Others	
C57BL/6J mice	Intraperitoneal	66 mg/kg	7 T	✓						Berkowitz et al., 2012
				✓						Berkowitz et al., 2015a
				✓						Giordano et al., 2015
	Subcutaneous osmotic pump	160 mg/kg/wk	4.7 T			✓		✓		Laine et al., 2017
C57BL/6J mice, C57BL/6J/129S6 mice, Opn4-/- mice	Intraperitoneal	66 mg/kg	4.7 T, 7 T	✓						Berkowitz et al., 2010, 2016
C57BL/6J mice, DBA/2J mice	Intraperitoneal	66 mg/kg	4.7 T	✓						Calkins et al., 2008
C57BL/6J mice, SOD1OE mice	Intraperitoneal	66 mg/kg	4.7 T	✓						Berkowitz et al., 2009a
Ca(v)1.4(-/-), Arr1(-/-) and Ca(v)1.3(-/-) C57BL/6J mice	Intraperitoneal	66 mg/kg	7 T	✓						Berkowitz et al., 2015b
C57BL/6J mice, GNAT1-/- mice	Intraperitoneal	66 mg/kg	7 T	✓						Berkowitz et al., 2014
Cav-1 KO C57BL/6J mice	Intraperitoneal	66 mg/kg	7 T	✓						Li et al., 2012
UM-HET3 mice	Intraperitoneal	66 mg/kg	7 T	✓						Berkowitz et al., 2017
rd1/rd1 mice	Intraperitoneal	66 mg/kg	4.7 T, 7 T	✓	✓					Ivanova et al., 2010
Abca4-/- Rdh8-/- double KO mice	Intravitreal	2.4 μL; 5 mM	7 T	✓						Schur et al., 2015
Sprague-Dawley rats	Intraperitoneal	44 mg/kg	4.7 T	✓						Berkowitz et al., 2006
				✓						Berkowitz et al., 2007a
				✓						Berkowitz et al., 2007b
		66 mg/kg		✓		✓	✓	✓		Bissig and Berkowitz, 2009
		0.2 mmol/kg						✓		Daducci et al., 2014
		50 mg/kg						✓		Kim et al., 2014
	Intravenous	88 mg/kg	11.7 T	✓						De La Garza et al., 2012
				✓						Muir et al., 2015
	Intracortical	100 nL; 500 mM	7 T					✓		Chan et al., 2014a
Sprague-Dawley rats, RCS rats	Intraperitoneal	44 mg/kg	4.7 T	✓						Berkowitz et al., 2008
Sprague-Dawley rats, Lewis rats	Intraperitoneal	44 mg/kg	4.7 T	✓						Berkowitz et al., 2007c
Sprague-Dawley rats, Long-Evans rats	Intraperitoneal	66 mg/kg	4.7 T, 7 T	✓		✓	✓	✓		Bissig and Berkowitz, 2011
		44 mg/kg		✓						Berkowitz et al., 2011
Long-Evans rats	Intraperitoneal	44 mg/kg	7 T	✓						Bissig et al., 2013
WAG/RijHs-rnu rats	Intraperitoneal	44 mg/kg	4.7 T	✓						Braun et al., 2007
Wistar rats	Intra-arterial or intravenous (+BBB opening)	5 mg/kg; 1.8 mL/min	3 T					✓		Fa et al., 2011


*If a study examined dose-dependent effects, the reported optimal dose was chosen (ON, optic nerve; SC, superior colliculus; LGN, lateral geniculate nucleus; VC, visual cortex; BBB, blood–brain barrier; KO, knock out).*

Manganese-enhanced MRI can detect layer-specific changes in retinal ion demand in response to light or dark adaptation. Upon systemic MnCl_2_ administration, healthy adult rats that were maintained in dark conditions showed higher signal intensity in the outer retinal layers, indicative of increased ion demand of photoreceptors when compared to rats maintained in light conditions (Berkowitz et al., 2006; De La Garza et al., 2012) (Figure 3C). Additionally, the inner retinal layers of light-stimulated rats had greater enhancements compared to the inner layers of dark-adapted rats, suggesting differential activity-dependent mechanisms of Mn^2+^ contrast across retinal layers (De La Garza et al., 2012). Such intraretinal MEMRI contrasts appear to be sensitive to melanopsin regulation (Berkowitz et al., 2010, 2016), horizontal cell inhibitory signaling (Berkowitz et al., 2015b), visual cycle activity (Berkowitz et al., 2009b), and channelrhodopsin-2-mediated activity *in vivo* (Ivanova et al., 2010). In addition, adult rats pretreated with diltiazem, a calcium channel antagonist, suppressed intraretinal Mn^2+^ uptake and MEMRI enhancement (Berkowitz et al., 2007b), indicative of the role of calcium channels in the Mn^2+^ uptake in the retina.

Manganese-enhanced MRI has been used to detect Ca^2+^-dependent abnormalities in aging, glaucomatous, and diabetic retinas (Calkins et al., 2008; Bissig et al., 2013; Berkowitz et al., 2014; Muir et al., 2015) (Figure 2K) as well as retinal ischemia/reperfusion (Berkowitz et al., 2008) and experimental retinopathy of prematurity (Berkowitz et al., 2007b, 2011). Calkins et al. (2008) demonstrated the use of MEMRI in identifying age-related ocular changes in hereditary glaucoma in a mouse model. Upon intraperitoneal MnCl_2_ injection, C57BL/6J mice at 10–11 months old demonstrated larger Mn^2+^ retinal uptake in the inner and outer retina by 18 and 24% respectively than young mice at 2–3 months old, whereas the aged and severely diseased DBA/2J glaucoma mice demonstrated a 6% decrease in inner retinal Mn^2+^ uptake and 5% increase in outer retinal Mn^2+^ uptake when compared to the younger and milder counterparts. Taken together, these findings indicate that MEMRI can be used to evaluate age-related activity changes and tracing the disease progression in glaucoma across retinal layers over an extensive period of time. On the other hand, a common animal model for diabetes involves the use of streptozotocin which is an alkylating antineoplastic agent toxic to insulin-producing beta cells of the pancreas. Several MEMRI experiments have utilized this model to characterize changes in neuronal integrity during the progression of this disease (Berkowitz et al., 2012, 2015a; Giordano et al., 2015; Muir et al., 2015; Kancherla et al., 2016). In brief, Mn^2+^-dependent activity in the outer retina decreased in dark conditions as the duration of hyperglycemia increased, suggesting altered ion homeostasis which is important in maintaining depolarizing dark current in photoreceptors when unstimulated. These changes were detected as early as 14 days after streptozotocin injection (Muir et al., 2015). However, MEMRI was not able to detect changes in the inner retina that were picked up by electroretinogram, which could mean that the two methods measure different aspects of neuronal activity or that MEMRI is not as sensitive with regards to detecting electrical changes in the diabetic inner retina. Using intraretinal MEMRI, loss of caveolin-1, an integral component of caveolar membrane domains involved in glaucoma, diabetic retinopathy, and autoimmune uveitis, was found to impair retinal function via disturbance of subretinal ion/fluid homeostasis (Li et al., 2012). MEMRI has an advantage of considerable spatial resolution when compared to electroretinography and hence the combination of MEMRI and electroretinogram may provide important insights into the spatiotemporal activity changes in future studies (Schur et al., 2015). One of the key etiological factors of dysfunctional rod photoreceptors in diabetic retinopathy involves oxidative stress (Berkowitz et al., 2015a). Hence, intraretinal MEMRI has been used to evaluate the effectiveness of anti-oxidative administration in reducing such damage (Giordano et al., 2015). Intraretinal MEMRI can also be used to examine other treatment regimens such as alpha-lipoic acid (Berkowitz et al., 2007c), 11-*cis*-retinal treatment (Berkowitz et al., 2009b, 2012), prophylactic retinylamine treatment (Schur et al., 2015), repetitive hypoxic-preconditioning (Berkowitz et al., 2008), and superoxide dismutase overexpression (Berkowitz et al., 2009a).

Manganese-enhanced MRI can also assess alterations in intraretinal ion demand due to ocular injury and tumorigenesis. Upon Na^+^/K^+^-ATPase inhibition by intravitreal ouabain injection, rats showed reduced retinal function as indicated by decreased receptor and post-receptor MEMRI signal intensities compared to phosphate-buffered saline-injected controls (Berkowitz et al., 2007a). In contrast, retinal pigment epithelium damage by intraperitoneal sodium iodate administration in rats resulted in increased levels of intraretinal Mn^2+^ uptake (Berkowitz et al., 2007a). Additionally, MEMRI has been shown to detect ocular tumor progression and its effects on non-tumor-bearing retinal cells. In a study supporting this notion, rat eyes were imaged at 14 days after implantation of C918 tumor cells into the suprachoroidal space. A cohort of these animals received intraperitoneal MnCl_2_ administration and showed a 30% increase in tumor signal intensity, a 17% increase in outer retinal signal intensity, but no change in inner retinal intensity in tumor bearing eyes when compared to rats injected with saline (Braun et al., 2007). This indicates the potential of MEMRI in imaging early oncogenic aspects of the visual system.

Cortical activity mediated through visual stimuli in awake and free-moving rats can also be imaged by MEMRI after systemic MnCl_2_ injection with or without temporarily opening the blood–brain barrier (Bissig and Berkowitz, 2009; Fa et al., 2011). Rats that received visual stimulation demonstrated significantly higher MEMRI signal intensities in layers IV and V of the primary visual cortex relative to the dark-adapted rats (Bissig and Berkowitz, 2009) (Figure 3C). Interestingly, the primary visual cortex also showed significantly more Mn^2+^ uptake after acoustic stimulation in rats, indicative of MEMRI detection of cross-modal brain activity (Kim et al., 2014). Given that MEMRI has no depth limitation, simultaneous detection of activity in both the eye and brain can be performed which allows for direct comparisons between retinal, subcortical, and cortical activities in the same sessions upon visual stimulation (Bissig and Berkowitz, 2011). Last but not least, MEMRI can help probe altered functionality in the visual cortex in animal models of psychiatric disorders such as chronic psychosocial stress (Laine et al., 2017) and interferon-α-induced depression (Daducci et al., 2014).

## Investigation of Glial Activity

Manganese-enhanced MRI has been shown to reflect glial activity changes within and beyond the visual system, yet the underlying mechanisms remain debatable. Some studies observed linkages between Mn^2+^ enhancement and astrocytic activity mediated by glutamate synthetase, manganese-superoxide dismutase (MnSOD), and calcium channels (Yang and Wu, 2007, 2008; Yang et al., 2008; Gadjanski et al., 2009; Hoffmann et al., 2013), whereas others observed increased Mn^2+^ uptake in relation to microglial activity (Haapanen et al., 2007; Wideroe et al., 2009, 2011). Oligodendrocytes may also uptake Mn^3+^ via the transferrin receptors, whereas Mn^3+^ is paramagnetic and can be converted from Mn^2+^ (Golub et al., 1996; Gunter et al., 2013). In this section, we will address various findings in the literature that attempted to identify and differentiate glial contributions to Mn^2+^ enhancement in MEMRI. A list of relevant MEMRI studies involving the visual system can be found in Table 4.

**Table 4:** Summary of MEMRI protocols used for identifying or differentiating glial activity in the visual system in terms of species, delivery route, Mn^2+^ dose, magnetic field strength and anatomical structures enhanced and studied.

Species	Delivery route	Mn^2+^ dose	Field strength	Anatomical structures of interest						Citation
					
				Retina	ON	SC	LGN	VC	Others	
Sprague-Dawley rats	Intraperitoneal	88 mg/kg; 100 mM; 15 μL/min	7 T				✓	✓		Yang and Wu, 2008; Yang et al., 2008
		45 mg/kg;100 mM	7 T				✓	✓		Chan et al., 2017
Wistar rats	Intraperitoneal	40 mg/kg; 100 mM	7 T				✓	✓		Wideroe et al., 2009, 2011
Brown Norway rats	Intravenous	50 μmol/kg	2.35 T		✓					Gadjanski et al., 2009
		20 mg/kg	9.4 T	✓	✓					Hoffmann et al., 2013


*If a study examined dose-dependent effects, the reported optimal dose was chosen (ON, optic nerve; SC, superior colliculus; LGN, lateral geniculate nucleus; VC, visual cortex).*

Several studies supported the use of MEMRI for the detection of astrocytic activity in the healthy brain or in response to neuronal injury. Within the gray matter, astrocytes produce glutamine synthetase, an enzyme which uses Mn^2+^ as a cofactor. After systemic Mn^2+^ administration to healthy rats, longitudinal MEMRI showed maximal T1-weighted enhancement at day 1 in brain structures that are known to possess high contents of unsaturated glutamine synthetase (Patel et al., 1985; Aschner et al., 1999) as well as high basal glutaminergic activity (Norenberg, 1979), both of which may cause uptake of the diffused Mn^2+^ leading to T1-weighted hyperintensity. In contrast, in brain regions with low glutamine synthetase contents such as the striatum, the T1-weighted signal enhancement appeared less pronounced at day 1 and peaked at a later time (Chan et al., 2017). Upon mild unilateral hypoxic-ischemic brain injury, the ipsilesional visual cortex and nearby brain regions were specifically enhanced by exogenous Mn^2+^ on T1-weighted imaging near the later stages of the disease (Yang and Wu, 2008) (Figure 3D). Interestingly, such enhancement patterns colocalized with areas of delayed neurodegeneration using immunohistochemical markers of glutamine synthetase and MnSOD (Yang and Wu, 2007, 2008). Since glutamine synthetase and MnSOD are manganese-binding enzymes that impart protection against glutamate excitotoxicity and oxidative stress, it is likely that the hyperintense Mn^2+^ signals in the injured brain tissues may be attributed to increased glutamine synthetase and MnSOD expression (Bidmon et al., 1998; Fujioka et al., 2003; Wang et al., 2006; Chan et al., 2017). MnSOD can be found in significantly higher amounts in the astroglia than in neurons, while glutamine synthetase is astrocyte specific which accumulates in gray matter lesions. Taken together, it is possible that Mn^2+^ may enhance astrocyte repair-related activity and aid in the detection of gray matter-related injuries (Yang and Wu, 2008; Yang et al., 2008).

It is important to note that the mechanisms of Mn^2+^ uptake and glutamine synthetase or MnSOD activity have not been thoroughly examined so caution is warranted in interpreting the results. There may be alternative pathways that can cause Mn^2+^ enhancement in astrocytes. For example, certain glial cells possess L-type voltage-operated calcium channels (Cheli et al., 2016), through which Mn^2+^ ions have been observed to pass (Bedenk et al., 2018). Knocking out Ca_v_1.2 channels in astrocytes decreases calcium influx by approximately 80% after plasma membrane depolarization and inhibits astrocyte activation, proliferation, and migration (Cheli et al., 2016). In contrast, Ca_v_1.2 channels were upregulated more in activated astrocytes compared to quiescent astrocytes (Cheli et al., 2016). In rats with optic neuritis, intravenous Mn^2+^ administration led to significantly stronger optic nerve enhancement as compared to their healthy controls (Gadjanski et al., 2009; Hoffmann et al., 2013). Such enhanced Mn^2+^ uptake from the systemic circulation to the injured optic nerve appeared to be mediated by calcium influx and calpain activation in the degenerating axons and/or during gliosis (Gadjanski et al., 2009; Hoffmann et al., 2013).

In addition to astrocytes, recent studies supported the notion that Mn^2+^ enhancement is associated with microglial activation (Wideroe et al., 2009, 2011). Wideroe and colleagues combined MEMRI with immunohistochemistry to characterize the detection of cellular changes of more severe hypoxic-ischemic brain injury in neonatal rats. Six hours after cauterization of the right carotid artery, rats were given Mn^2+^ intraperitoneally for MEMRI scans on days 1, 3, and 7, and brain tissue samples were taken for immunohistochemical staining using CD68 for activated microglial cells and glial fibrillary acidic protein for reactive astrocytes. On days 1 and 3, widespread reactive astrocytes were found across the injured hemisphere, whereas only a few activated microglial cells were observed on day 1 across the ipsilesional hemisphere. By day 7, the number of activated microglial cells increased along with increasing CD68 staining intensities, and many of these cells were concentrated in the dorsolateral thalamus near the lateral geniculate nucleus (Wideroe et al., 2009). MEMRI scans revealed small spots of high signal intensities in the thalamus on day 3, and later in large parts of the dorsolateral thalamus and parts of the hippocampus and remaining cortex on day 7 (Wideroe et al., 2009). At day 7, two animals with low density of activated microglia in the thalamus also had almost no detectable Mn^2+^ enhancement in T1-weighted imaging. Among the immunohistochemical markers, activated microglia on day 7 had the best spatial agreement with the MEMRI scans. It is possible that microglia activation increased reactive oxygen species and the release of glutamate which upregulated the expression of MnSOD and glutamine synthetase in astrocytes, resulting in delayed Mn^2+^ enhancements on day 7. However, the enhancements of another animal group which received MnCl_2_ 6 days after injury never reached the intensity of those which received MnCl_2_ within hours after the injury. Such signal intensity differences indicate that the Mn^2+^ enhancements in animals receiving MnCl_2_ early after injury may have been the result of Mn^2+^ accumulation in microglia over the course of several days as opposed to active uptake (Wideroe et al., 2009). This evidence suggests other potential mechanisms of Mn^2+^ uptake in glial cells, which further supports the need for caution when interpreting Mn^2+^ enhancements.

Apart from glial activity detection, there are some initial attempts to distinguish between glial and neuronal activity in inflammatory and degenerative diseases using MEMRI. After exposure to known pro-inflammatory agents via intracranial lipopolysaccharide injection (Bade et al., 2013), glial inflammation appeared to enhance neuronal activity and neuronal Mn^2+^ uptake. Glial Mn^2+^ content, however, was independent of the state of glial cells. These results bolster the thought that MEMRI reflects neuronal excitotoxicity and related impairments such as neuroinflammation. Other investigators also probed into the relation between MEMRI signal enhancement and glial reaction in different disease models including cathepsin D deficiency (Haapanen et al., 2007), epilepsy (Immonen et al., 2008), hyperoxia (Morken et al., 2013), and prenatal X-ray exposure (Saito et al., 2012) in both gray and white matters. Some studies revealed that elevated MEMRI signal enhancement tends to co-localize with activated microglia (Haapanen et al., 2007), reactive astrocytes (Kawai et al., 2010) or both (Wideroe et al., 2009, 2011; Morken et al., 2013), while one study demonstrated a negative correlation between Mn^2+^-enhanced brain tissue R1 changes and density of reactive astrocytes (Saito et al., 2012). Since R1 is a potential marker of Mn^2+^ uptake (Chuang et al., 2009), this investigation suggested that astrocytic reaction suppresses rather than increases Mn^2+^ signal enhancement. Also, Immonen et al. (2008) found no correlation between change in MEMRI signal and astrocyte activation status in the epileptic rat hippocampus. These varying findings can be attributed to different disease models and brain targets investigated, but also indicate that certain aspects of Mn^2+^ based imaging are yet to be deciphered, such as any concurrent changes in overall cell density (Saito et al., 2012) or axonal density (Immonen et al., 2008).

It is important to note that the diseases processes examined in the above studies are complex and are associated with a multitude of molecular, cellular, and pathophysiological changes which tend to obscure the clear findings about glial activation. These processes include cellular edema, necrosis (Wideroe et al., 2009, 2011; Morken et al., 2013), and apoptosis (Haapanen et al., 2007). Once these events ensue, they tend to impact the neuronal activity leading to alterations in neuronal Mn^2+^ uptake thereby masking the interpretations of MEMRI signal enhancement. In an attempt to differentiate these degenerative events, one study probed the spatiotemporal MEMRI profiles upon Ca^2+^ dysfunction after traumatic brain injury (Talley Watts et al., 2015). The areas of controlled cortical impact displayed a distinct biphasic profile with MEMRI hyperintensity at hour 1–3 and gradual loss of intensity on days 2–14, while no such biphasic effect was seen in the vehicle group. A hyperintense area was also observed surrounding the impact core on days 7–14. Compared with immunohistochemistry, the MEMRI signal void in the impact core and the hyperintense rim on days 7–14 corresponded to tissue cavitation and reactive gliosis, respectively (Talley Watts et al., 2015). The authors concluded that MEMRI could identify excitotoxic injury during the hyperacute phase that precedes vasogenic edema. Hence, MEMRI can be employed as a complementary technique to conventional MRI to differentiate early stages of gliosis. In another *in vivo* rat study using combined systemic and stereotactic MEMRI to specifically track the cellular responses of astrocytes and neuronal pathways, a positive temporal correlation was reported between astrogliosis and the recovery of neuronal pathways at the chronic stage after stroke. Microglia, however, did not contribute to systemic MEMRI enhancement because they remained in the lesion core (Hao et al., 2016). While MnCl_2_ can stimulate the microglia dose-dependently (Zhang et al., 2007), it is also possible that systemic Mn^2+^ administration at low doses could exert anti-oxidative effects to some extent and preserve brain tissues at remote sites from delayed secondary damage (Singh et al., 1992; Hussain and Ali, 1999; Chan et al., 2008a, 2017). There are currently a number of approaches to mediate neurodegeneration via targeting astrocytes and microglia (Yun et al., 2018; Liddelow and Sofroniew, 2019; van der Merwe et al., 2019). With better understanding of the Mn contrast mechanisms and careful interpretation, the combined Mn administration and MRI detection approaches may be useful for future investigations of post-injury cellular events and functional reorganization.

## Limitations

While MEMRI possesses considerable potential for evaluating the structure and function of the retina, optic nerve, and visual brain connections, there are important limitations that need to be addressed. For example, while Mn^2+^ enhancement is generally interpreted as the surrogate of the tract viability and neuronal activity, this explanation may not always be accurate because mice with genetic retinal blindness were found to give MEMRI enhancements in the optic nerve after intraocular Mn^2+^ administration (Bearer et al., 2007). Manganese-enhanced MRI likely reflects both activity-independent uptake at base levels as well as activity-dependent uptake upon stimulations or interventions (Wang L. et al., 2015). Another important feature to consider is the depth of anesthesia and body temperature during dynamic MEMRI, both of which may alter neuronal transport (Aoki et al., 2002; Smith et al., 2007). Apart from age-related changes in neuronal Mn^2+^ transport (Minoshima and Cross, 2008; Chan et al., 2012a), there are age-related changes in the blood–ocular and blood–brain barriers (Berkowitz et al., 1998; Minoshima and Cross, 2008; Chan et al., 2012a; Qiu et al., 2018), which have direct bearing on the bioavailability of Mn^2+^ in the neural tissues. Thus, the same Mn^2+^ dose and protocol may give different MEMRI results in animal models of varying ages.

To date, the biochemical metabolome of Mn^2+^ within the brain is poorly understood; therefore, the interpretations of the results obtained by MEMRI remain rather speculative. One of the proposed mechanisms of Mn^2+^ transport along the neuronal tracts is through kinesin-mediated vesicular transport. However, in kinesin light chain 1 knockout mice, Mn^2+^ transport was slowed but not halted, while Mn^2+^ enhancement after 24 h was similar to normal mice. This suggests that kinesin is not essential to Mn^2+^ transport and that other mechanisms may be involved (Bearer et al., 2007). Additionally, the directional interpretation of Mn^2+^ transport along the tracts may not be as accurate as is generally thought. For example, although fast anterograde axon transport of Mn^2+^ is commonly observed, retrograde transport can occur concurrently albeit at a slower rate (Matsuda et al., 2010). Experimental modeling from dynamic MEMRI also noted the potential involvement of a wide range of apparent Mn^2+^ transport rates along the optic nerve (Olsen et al., 2010), whereas the contributions of neuronal and glial activity to MEMRI enhancement appear multifaceted. Due to the lack of specificity in our understanding of Mn^2+^ transport, the interpretations of MEMRI observations can range from passive diffusion to active transport along microtubules through vesicular traffic, membrane integrity, ion integrity, calcium channel activity, enzyme activity, cellular or axonal density, synaptic function, and the combinations thereof.

Currently, the major drawback for MEMRI use is the toxicity of MnCl_2_, especially at high concentrations, which limits its use in humans. Table 5 provides details about Mn^2+^ toxicity within and beyond the visual system upon local and systemic Mn^2+^ manifestations. For example, Mn^2+^ injection into the mouse eyes disrupts the electrical response to light in the visual system. At high volumes (>0.25 μL) of injectate to mouse eyes there is a volumetric effect. At low volumes (<0.25 μL of 200 mM) a transient effect on visually evoked potentials which reverses after 24 h was observed with Mn^2+^ but not with saline (Bearer et al., 2007). Even at these low amounts, a permanent loss of 10–20% of the axons in the optic nerve was found (Bearer et al., 2007). Similar effects on retinal morphology and visual responses were also observed upon topical Mn^2+^ administrations in the mouse eyes (Sun et al., 2012; Lin et al., 2014a) and upon intravitreal Mn^2+^ administrations in the rabbit eyes (Zhang et al., 2010). Hence Mn^2+^ exposure may have long-term effects on neurons. Apart from neurotoxicity, Mn^2+^ may manifest as cardiotoxicity when given systemically at high doses leading to changes in heart rate, cerebral blood flow, parasympathetic tone, and eventually brain functions also. While there is no evidence that intraperitoneal doses of MnCl_2_ in the range from 44 to 66 mg/kg alter histology, visual performance, blood–barrier integrity, visual behavior or electrophysiology (Berkowitz et al., 2006, 2007a, 2015b; Bissig and Berkowitz, 2011; Bissig et al., 2013), it is important to mention that fractionation does not completely avoid the toxic effects of Mn^2+^ if the dosages of the fractions are high. Paradoxically, keeping the dosage low necessitates multiple administrations which increase the risk of injection injuries. Severe necrosis have been reported to occur near the injection site on the tails of animals administered with fractions of 90 mg/kg (Bock et al., 2008). Skin ulceration was also noted in mice receiving high MnCl_2_ doses via subcutaneous osmotic pumps (Vousden et al., 2018).

**Table 5:** Summary of toxic effects observed in the visual system and beyond after high-dose Mn^2+^ use in MEMRI.

Species	Delivery route	Citation	Mn^2+^ dose	Frequency	Toxicity
C57BL/6J mice	Intravitreal	Bearer et al., 2007	0.125 μL; 50 mM	Single dose	Reduced amount of response to light 4 h post-injection, full recovery 24 h post-injection, 10–20% decrease of axons in the optic nerve.
			0.25 μL; 200 mM		Reduced amount of response to light 4 h post-injection, some function returned 24 h post-injection, optic nerve diameter on injected side was 6% smaller than non-injected side, approximately 25% decrease in axons per unit area in optic nerve.
			0.5 μL; 200 mM		No response to light in both eyes due to volumetric effect of injection 4 h post-injection, small potential change 24 h post-injection, optic nerve diameter on injected side was 6% smaller than non-injected side, approximately 25% decrease in axons per unit area in optic nerve.
		Haenold et al., 2012	2 μL; 50 nmol	Single dose	Retinal ganglion cell (RGC) density reduced by 21.5%, outer nuclear layer (ONL) barely detectable.
			2 μL; 100 nmol		Retinal ganglion cell density reduced by 20.7%, ONL not detectable, visual acuity drastically reduced.
	Topical	Sun et al., 2012	5 μL; 1 M	3x–7x/every 2 weeks	20–40% RGC loss, with corneal thickening and increased corneal opacity at 7x/every 2 weeks.
		Lin et al., 2014a	60 μL; 500 mM	Single dose	Slight drop in visual acuity 1 day after loading with recovery to normal at day 2.
			60 μL; 750 mM		Drastic drop invisual acuity followed by gradual recovery to normal range by day 5. Slight retinal swelling at day 1 with recovery to normal thickness at day 5.
			60 μL; 1 M		Loss of visual acuity, significant retinal swelling (∼26% increase) at day 1, and significant retinal thinning (∼31%) at day 7.
			0.25 μL; 200 mM		Slight retinal swelling at 4 h with recovery by 24 h.
	Intracameral	Lindsey et al., 2013	1.0 μL; 50 nmol	Single dose	Enlargement of spaces among collagen fibrils within corneal stroma after 1 week.
			1.0 μL; 100 nmol		Cataracts, outer plexiform layer (OPL) thinning in peripheral retina after 1 week.
			1.0 μL; 300 nmol		Cataracts, significant OPL thinning, loose matrix/inflammatory cells in anterior chamber, 125% increased cells in inner plexiform layer (IPL) after 1 week.
			1.0 μL; 500 nmol		Cataracts, significant OPL thinning, loose matrix/inflammatory cells in anterior chamber, 163% increased cells in IPL, absent retinal nerve fiber layer after 1 week.
	Subcutaneous osmotic pump	Vousden et al., 2018	50 mg/kg/day	Continuous	Skin ulceration.
Fischer rats	Intravitreal	Thuen et al., 2008	3 μL; 300, 1500, 3000 nmol	Single dose	12%, 57%, and 94% reduced RGC density respectively; swelling of the globe, cataracts, corneal opacities, anterior and posterior chamber hemorrhages, retinal degeneration (1500–3000 nmol); failed clearance of Mn^2+^ from vitreous (3000 nmol).
Sprague-Dawley rats	Intravitreal	Luo et al., 2012	2 μL; 25 mM	Single dose	Increased numbers of ribosomes.


			2 μL; 50 mM		RGC outer segment and retinal pigment epithelial microvilli damage, RGC mitochondrial cristae disorganization and ribosomal disaggregation.
			2 μL; 75–150 mM		Vacuoles in RGCs and outer segments of photoreceptors.
			2 μL; 300 mM		Vacuoles in RGCs and outer segments of photoreceptors, near complete loss of outer plexiform layer, retinal thinning, complete destruction of RGC outer segment, severe RGC retinal pigment epithelial microvilli damage.
		Xiao et al., 2019	2 μL; 100 or 200 mM	Single dose	Nerve tract edema, dendrite and axon swelling and fiber loss in LGN, astroglial swelling in superior colliculus (100 and 200 mM) and visual cortex (200 mM).
	Intraperitoneal	Bock et al., 2008	3 mg/kg × 60 mg/kg; 25 mM; 1.25 ml/h	Fractionated dose	Early weight loss.
	Intravenous	Bock et al., 2008	180 mg/kg; 100 mM	Single dose	Early weight loss, abdominal induration followed by substantial bleeding inside the abdominal wall, hunched and lethargic, potential heart failure, tail necrosis.
	Intrathecal	Liu et al., 2004	25 μL; 25 mM	Single dose	Transient respiratory and cardiac distress immediately after injection resolved after 20 min, acute ataxia during recovery, weight loss for 2–3 days.
			25–50 μL; 50 mM		67–83% of animals died within first 6 h.
New Zealand rabbits	Topical	Chen et al., 2016	400 μL; 50 mM	9x/every 5 min	Corneal edema in epithelium-removed group only, with alleviation by day 14.
			400 μL; 100 mM		Corneal edema in epithelium-removed group only.
			400 μL; 200 mM		Corneal edema present in epithelium-removed and epithelium-intact subgroups at days 1 and 14; corneal endothelium severely damaged.
Pigmented rabbits	Intravitreal	Zhang et al., 2010	25 μL; 10–40 mmol/L	Single dose	Reversible reduction in flash electroretinogram b-wave amplitude at ≤15 mmol/L, irreversible damages in retinal function and morphology at ≥20 mmol/L.


*The corresponding species, Mn^*2*+^ delivery route, dosage and frequency are also noted. For each study cited, only doses with observed toxic effects were included in the table.*

## Future Directions

One of the most important aspects for the future of Mn^2+^ based imaging is to identify less toxic Mn^2+^ salts that can be used as contrast agents for both clinical and preclinical applications. This will avoid the design handicap that we witness in the current protocols including systemic toxicity and local injuries to the target tissues and the injection sites. A potential solution is the use of chelating agents designed to sequester Mn^2+^ ions and drive a slow-release in biological systems. Mangafodipir trisodium (MnDPDP, TeslaScan) is an example of such. This compound is consisted of Mn^2+^ ions and fodipir (dipyridoxyl diphosphate) as a chelating agent, and was approved for clinical use for pancreatic and hepatobiliary imaging (Lim et al., 1991; Rofsky and Weinreb, 1992), myocardial infarction detection (Pomeroy et al., 1989; Saeed et al., 1989), and diagnostics of cancer including hepatocellular carcinoma (Sutcliffe et al., 2011). Initial studies involving intravenous MnDPDP administrations to healthy human volunteers showed maximal T1-weighted MRI enhancement within 15–30 min in the liver, pancreas, spleen, and kidney that lasted for hours (Wang et al., 1997) until clearance from hepatocytes within 24 h of administration (Koh et al., 2007). MnDPDP has been demonstrated not to cause adverse injection site injury or dermal hypersensitivity (Larsen and Grant, 1997). Also, *in vivo* catecholamine release triggered by MnDPDP administration prevents the potentially negative ionotropic effects of Mn^2+^ ions on cardiac function (Jynge et al., 1997). The reduced initial cardiac uptake of Mn^2+^ may account for the favorable cardiovascular safety of MnDPDP (Ni et al., 1997) with low incidence of serious side effects (Federle et al., 2000).

With regards to the visual system, intravitreal MnDPDP injection allows MEMRI tract tracing in the rat retina and optic nerve similar to intravitreal MnCl_2_ injection (Olsen et al., 2008), while systemic MnDPDP administration allows MEMRI detection of dark- or light-adapted retinal function at a clinically relevant dose in healthy rats (Tofts et al., 2010). MnDPDP enhancement may also reflect gliosis in hypoxic-ischemic injured rat brains in T1-weighted imaging (Yang and Wu, 2009). While the MRI-contrast properties of MnDPDP rely on the release of Mn^2+^, MnDPDP possesses MnSOD-mimetic activity which is dependent on the manganese ions that are bound to the fodipir part (Karlsson et al., 2015). MnDPDP may also help identify populations of neural stem and progenitor cells within the intact embryo brain for investigating neurodevelopment and disease mechanisms (Norris et al., 2013). On a negative note, MnDPDP has been shown to induce skeletal abnormalities in fetal rodents thereby raising a caveat for the toxicity of teratogenic nature (Grant et al., 1997). It is important to note that MnDPDP does not pose much toxicity in animal models unless it is used in high doses (Elizondo et al., 1991). Though MnDPDP-based neuroimaging has found remarkable use in rodent models in recent years, its use in human brains remains limited given the lack of thorough toxicity, sensitivity, and specificity assessments at relevant doses (Wang et al., 1997). Human eyes are at least 3–7 times bigger than rodent eyes in diameter (Van Cruchten et al., 2017), while the human brains are more than a 1000 times larger than rodent brains in volume (Semple et al., 2013). Also, rodents do not have lamina cribrosa or macula, both of which play important roles in vision disorders such as glaucoma and age-related macular degeneration. For more accurate determinations of human visual neurophysiology and visual disease mechanisms, we may likely see over the coming decade the translation of MnDPDP neuroimaging from rodents toward larger mammals before practical applications to the human visual system.

Apart from Mn^2+^ safety, one of the essential goals for future MEMRI studies would be to decipher the metabolic arena (Barandov et al., 2019) and transport of Mn^2+^ in the neuronal milieu as that would aid in developing better studies and understanding not only structure and function but also mechanism of disease progression and protocols for differential diagnosis and prognosis. Employment of nanotechnology such as nanoparticle-based delivery of Mn^2+^ may deliver Mn^2+^ to the eye and the visual pathway more specifically without altering the blood–ocular or blood–brain barrier (Bertin et al., 2009; Alaverdashvili et al., 2017; Sanchez-Ramos et al., 2018; Barandov et al., 2019). Additionally, sustained release of Mn^2+^ ions can improve Mn^2+^ entry duration and MEMRI sensitivity while minimizing toxicity and injection frequency (Bertin et al., 2009; Morch et al., 2012). MRI hardware and imaging sequence developments may also pave way to improve MEMRI sensitivity for detecting spare neuronal connectivity or weak brain activity change. Future studies may utilize real-time MRI to allow monitoring the outcomes of pharmacological interventions in a dynamic environment (Lu et al., 2008). Awake dynamic MEMRI may also be used for probing brain activity and pharmacological kinetics while minimizing confounds from anesthetic regimes (Febo et al., 2005; Schroeder et al., 2016).

In summary, MEMRI has considerable potential for unveiling the neural circuits of the visual system in relation to development, impairments, plasticity, and restoration. There are many unknown functional and disease processes involving the visual system that may be deciphered with the help of MEMRI. However, more work has to be done in regard to understanding precisely how Mn^2+^ is distributed in biological systems while avoiding the pitfalls of Mn^2+^ toxicity. Uncovering the mysteries behind Mn^2+^ detection mechanisms is the key to opening new doors for MEMRI as a non-invasive and useful *in vivo* tool in studying different aspects of ophthalmology and visual neuroscience.

## Data Availability

No datasets were generated or analyzed for this study.

## Author Contributions

WD, MF, CL, and VA wrote the manuscript. KC proposed the topic and edited the manuscript.

## Conflict of Interest Statement

The authors declare that the research was conducted in the absence of any commercial or financial relationships that could be construed as a potential conflict of interest.
